# Fucoidans inhibit the formation of post-operative abdominal adhesions in a rat model

**DOI:** 10.1371/journal.pone.0207797

**Published:** 2018-11-21

**Authors:** Alex J. Charboneau, John P. Delaney, Greg Beilman

**Affiliations:** 1 University of Minnesota Medical School, Minneapolis, Minnesota, United States of America; 2 Department of Surgery, University of Minnesota, Minneapolis, Minnesota, United States of America; Seoul National University College of Pharmacy, REPUBLIC OF KOREA

## Abstract

**Purpose:**

Fibrin clot is essential for post-operative abdominal adhesion formation. Fucoidans, sulfated polysaccharides, inhibit fibrin clot formation. In addition, they inhibit inflammation and fibrosis, which also play important roles in adhesion formation. The purpose of this study was to evaluate fucoidans’ potential for inhibiting post-operative abdominal adhesions and measure their effects on systemic coagulation parameters when administered intraperitoneally (IP).

**Methods and materials:**

Female Sprague Dawley rats were studied. A 2.5x2.5cm full thickness segment of abdominal wall was excised. The skin edges were approximated. This model induces extensive adhesions and allows objective quantitation. Three fucoidans were evaluated- Sigma Fucoidan Crude (SFC), *Fucus vesiculosis 95%* (Sigma) and, Peridan. One protocol involved continuous infusion into the abdomen from a subcutaneous osmotic pump. Alternatively, boluses of the solutions were injected IP at the end of the operation. Rats were sacrificed a week later. Adhesion extent was scored. Systemic coagulation effects of fucoidans were also evaluated. INR and aPTT were measured following IP injection of the fucoidan solutions and after 7 days of continuous infusion.

**Results:**

Animals given a continuous infusion of either SFC or Peridan yielded adhesion reduction of 80 to 90% from control. Bolus Peridan had no discernable influence on adhesion formation, but a single bolus of SFC caused significant adhesion reductions. Peridan resulted in prompt aPTT elevations which fell to nearly normal by 5 hours. The maximum peak value after SFC injection was seen in 15 hours. The maximal INR elevations were around 2. Measurement of INR and aPTT after a week of continuous infusion of either Peridan or SFC, were always in the normal control range. The third agent, Sigma, frequently yielded intraperitoneal infection found at autopsy.

**Conclusions:**

These findings indicate that selected fucoidans infused intraperitoneally for a week after abdominal operations reduce adhesion extent by up to 90%.

## Introduction

Adhesions following abdominal or pelvic operations occur in the great majority, 65% to 95%, of cases. Adhesive intestinal obstruction later develops in as many as 15% of such individuals and requires operative relief in almost a third of these [1, 2]. Adhesions are responsible for up to 20% of infertility cases [3]. A common after-effect of pelvic operations is chronic pain, thought to be due to adhesions. Adhesions impede later abdominal operations by interfering with exposure and by increasing the risk of inadvertent enterotomies.

Beginning in the early 1900s, the quest began for means to prevent post-operative abdominal adhesions [4, 5]. Subsequent publications number in the thousands. Yet, today, only 3 commercial products have been approved by the FDA for this purpose, Adept (Baxter International), Interceed (Johnson and Johnson), and Seprafilm (Genzyme Corporation).

Numerous excellent reviews have been published on the subject [1, 6, 7], most of which address the issue of prevention. The reviews generally end with a statement to the effect that, we have learned much about the pathogenesis of postoperative adhesions, but that there is no convincing evidence that any currently available agents actually provide clinical benefit. Cochrane meta-analyses [8, 9, 10] similarly conclude that there is no useful fluid borne pharmacologic agent for preventing post-operative abdominal adhesions. Clinically used barrier methods of preventing adhesions have yielded modest reduction in adhesions and no known reduction in adhesion-related bowel obstruction to date [9, 11–14]

The conduct of an abdominal operative procedure involves inevitable and unpredictable peritoneal injuries remote from the surgical site, leading to adhesions. Omentum, which is highly mobile, participates in random patterns of adhesion formation. Protection of these scattered sites requires agents which can circulate throughout the abdomen to bathe its surfaces. It should be noted that fluid placed anywhere in the peritoneal cavity does circulate throughout [15].

The sequence of events leading to adhesion formation is well documented. Injury to the peritoneum generates an inflammatory reaction with accompanying exudation of fibrin-rich plasma and leukocytes, initially polymorphs. In short order monocyte-derived fibrocytes invade the fibrin clot. The resulting matrix serves to hold two injured surfaces in approximation, providing a scaffold for conversion of fibrin to a collagen adhesive attachment.

In 2011, Springate, Cashman, and colleagues submitted patent applications regarding Fucoidans for adhesion prevention [16, 17]. These documents include extensive experimental data, much of which is not to be found in the scientific literature. This group of investigators tested a total of 75 potential anti-adhesion agents in rat and rabbit models, including various fucoidan preparations. Their fundamental conclusion was that fucoidans were the most effective anti-adhesion agents among the many candidates tested. Fucoidans had not been previously reported as abdominal antiadhesive agents. Their efforts were first published as an abstract which described promising results from the use of a single intraperitoneal (IP) dose of a fucoidan solution in a rat cecal sidewall model [18].

A subsequent publication described excellent adhesion reductions in a different rat cecal sidewall model. Here the fucoidans were embedded in a hyaluronic glycerol film which was interposed between the injured cecum and the sidewall defect and so served additionally as a mechanical barrier [19]. These investigators also reported successful adhesion reductions in two rabbit uterine horn models [20]. Importantly, they found no significant toxicities, gross or biochemical, in any of their multiple different fucoidan studies, whether the agents were applied as a circulating fluid or fixed in a barrier material.

## Materials and methods

### Animals

This study was approved by the University of Minnesota Institutional Animal Care and Use Committee (IACUC). The protocol was approved by IACUC (Protocol Number: 1101A95274). Mature, female Sprague-Dawley rats, weighing between 300 and 400 grams, were obtained from Harlan Laboratories (Madison, WI). The animals were cared for by the University of Minnesota Research Animal Resources Department in accordance with the principles in the NIH Guide for Care and Use of Laboratory Animals [21]. They were acclimated for at least a week before entering the study. Euthanasia was accomplished with carbon dioxide asphyxiation or exsanguination from the heart under anesthesia.

### Agent preparation and delivery

Sigma Fucoidan Crude (SFC), purchased from the Sigma-Aldrich Corporation as a powder, had been extracted from *Fucus vesiculosis*. Peridan Concentrate solution, supplied by ARC Medical Devices, was derived from *Laminara japonica* and in a concentration of 50 mg/ml. Doses were chosen empirically based, in part, on the ranges reported in company literature or in patents. For each fucoidan these studies involved dose escalation seeking a level to inhibit adhesions without causing bleeding. An Alzet 2ML1 osmotic pump (Durect Corporation, Cupertino, CA) was used for the continuous perfusion studies. This model is calibrated to deliver 2 ml of liquid at a rate of 10 microliters per hour over the course of seven days. The pumps were aseptically prepared prior to implantation in a subcutaneous pocket. A 5 cm length of 5Fr PVC Feeding Tube (Covidien, Mansfield, MA) served as the conduit for delivery into the abdomen. The tube was filled with the drug solution at the outset to ensure that delivery would start promptly. SFC powder was dissolved in sterile water and diluted such that the intended one-week dose was contained in a 2cc volume. This solution was centrifuged for 10 minutes, leaving a small plug of visible debris. Supernatant was withdrawn and 2cc injected into the pump. Peridan Concentrate solution was diluted with water to get the chosen one-week dose of fucoidan in 2cc volume.

### Experimental model

The rat adhesion model utilized in all the present experiments was described by Gaertner [22] and has been used extensively in our lab. Anesthesia was induced with inhaled Isoflurane (5%) and oxygen (1 L/min) and maintained for the duration of the operation. The surgical site was aseptically prepared, and a 3 cm abdominal midline skin incision was made. A 2.5 by 2.5 cm full thickness square of abdominal muscle, fascia, and peritoneum was excised, preserving the skin. Hemostasis was achieved using clamps or digital pressure to avoid suture or cautery injuries. A second transverse incision was made in the right flank and a subcutaneous pocket was developed with the pump placed therein. The delivery catheter, tunneled through the abdominal wall, protruded about 2–3 cm into the abdomen with the tip lying in the right lateral gutter. One control group had no pump. A second control group got a continuous one-week water infusion. Skin incisions were closed with a single layer of running 4–0 polyglactin 910 suture. In this way, the subcutaneous layer, as well as the defect edge, cut fascia, muscle and peritoneum, were exposed to the abdominal viscera. Postoperative analgesia (Ketoprofen 5 mg/kg) was given for two days.

For single bolus experiments the operative preparation was identical, but no pump was employed. Two cc containing the chosen dose of fucoidan was injected into the abdomen as the incision was being closed.

### Adhesion studies

**Fig 1:**
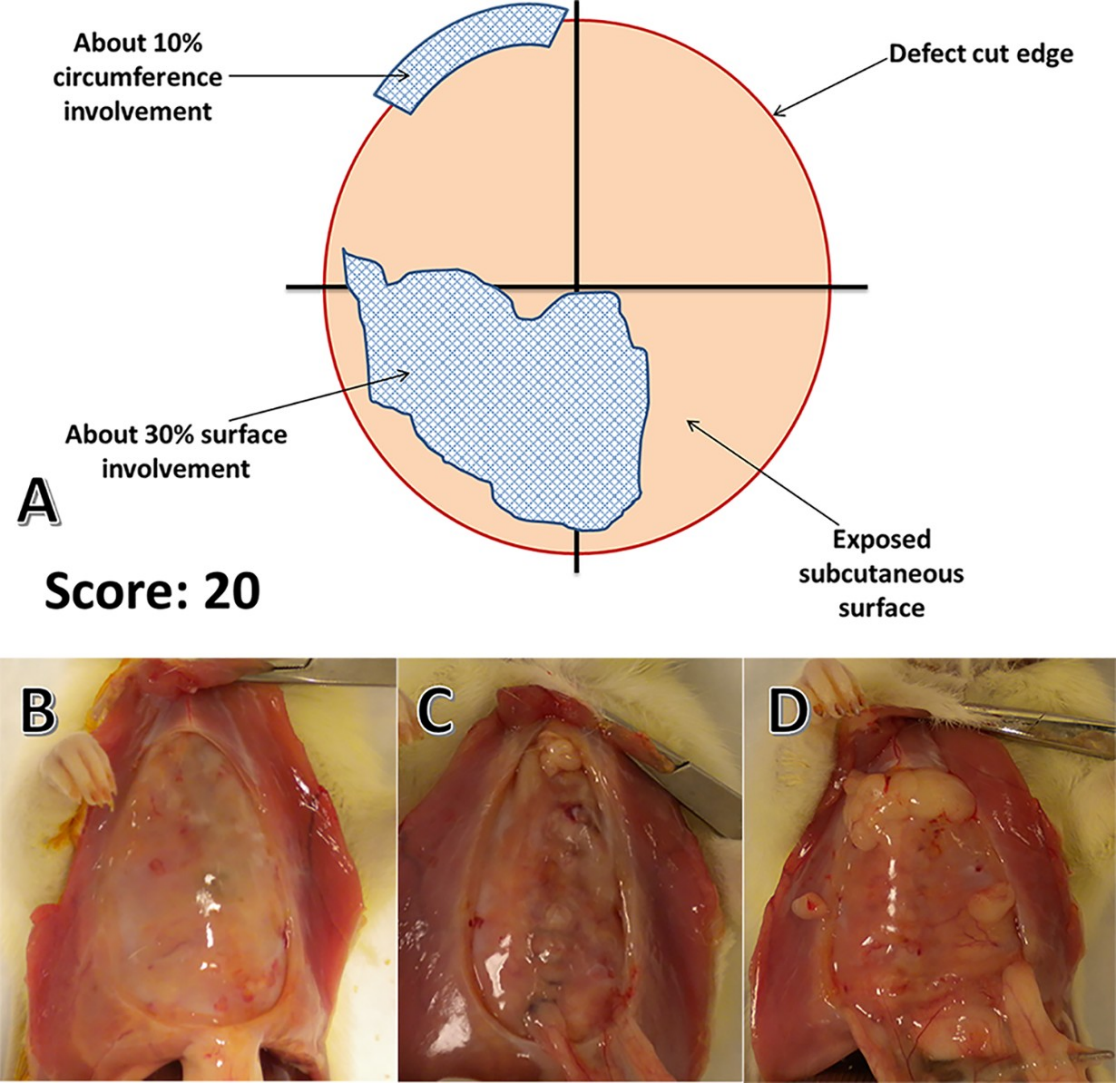
Diagram describing score derivation for each animal. The red circle represents the full thickness defect cut edge and the beige fill represents the sewn together subcutaneous tissue overlying the defect. Black lines were visualized by the scorer to assist quantifying percent adhesion coverage (A). Figure displays an adhesion score of 20. Representative photos of adhesion scores include 0 (B), 7.5 (C), and 45 (D).

Animals were sacrificed after 7 days. Gross observations regarding the general health as well as animal weight were recorded daily. Following euthanasia, a U-shaped incision was made extending from the suprapubic area bilaterally to the costal margins to fold back the ventral abdominal wall and expose the midline defect. The defect was visually divided into four quadrants to facilitate the scorer’s estimation of the percent of exposed subcutaneous tissue covered by adhesions. Adhesions to the defect cut edges were recorded as the estimated percent of the circumference involved. The percentage of the defect surface and percentage of edge circumference were averaged to provide a composite score (0–100). The field was photographed for later review (Fig 1). The scorer was not blinded.

The pump was removed and weighed. Any solution remaining was expelled by compressing the outer casing with pliers and then reweighing the pump. Results were thereby calculated and expressed based on the actual delivered dose of fucoidan. The total volume delivered was generally 1.5 to 1.7 cc. or 75–85% of the original dose of fucoidan.

### Coagulation studies

Systemic coagulation parameters, International Normalized Ratio (INR) and Activated Partial Thromboplastin time (aPTT), were measured after intraperitoneal injections of 50 mg of either of the fucoidan solutions, two hours after injection and at intervals thereafter up to 40 hours. Blood was obtained from anesthetized animals by terminal cardiac puncture. Systemic coagulation values were similarly assayed at the time of sacrifice in the rats that had received continuous infusions of IP drug or water for a week. Control values were established by obtaining blood from rats that had received no drugs.

### Statistical analysis

Statistical differences within the adhesion inhibition data were determined using single factor one-way analysis of variance (ANOVA) tests. *P <* 0.05 was considered evidence of significance between experimental groups. Post-hoc analysis was done using two-tailed Student’s t-tests assuming unequal variance. These calculations were performed using Microsoft Excel 2010 Data Analysis Tools (Microsoft Corporation, Redmond, WA).

## Results

### Adhesion reduction after continuous IP infusion

**Fig 2:**
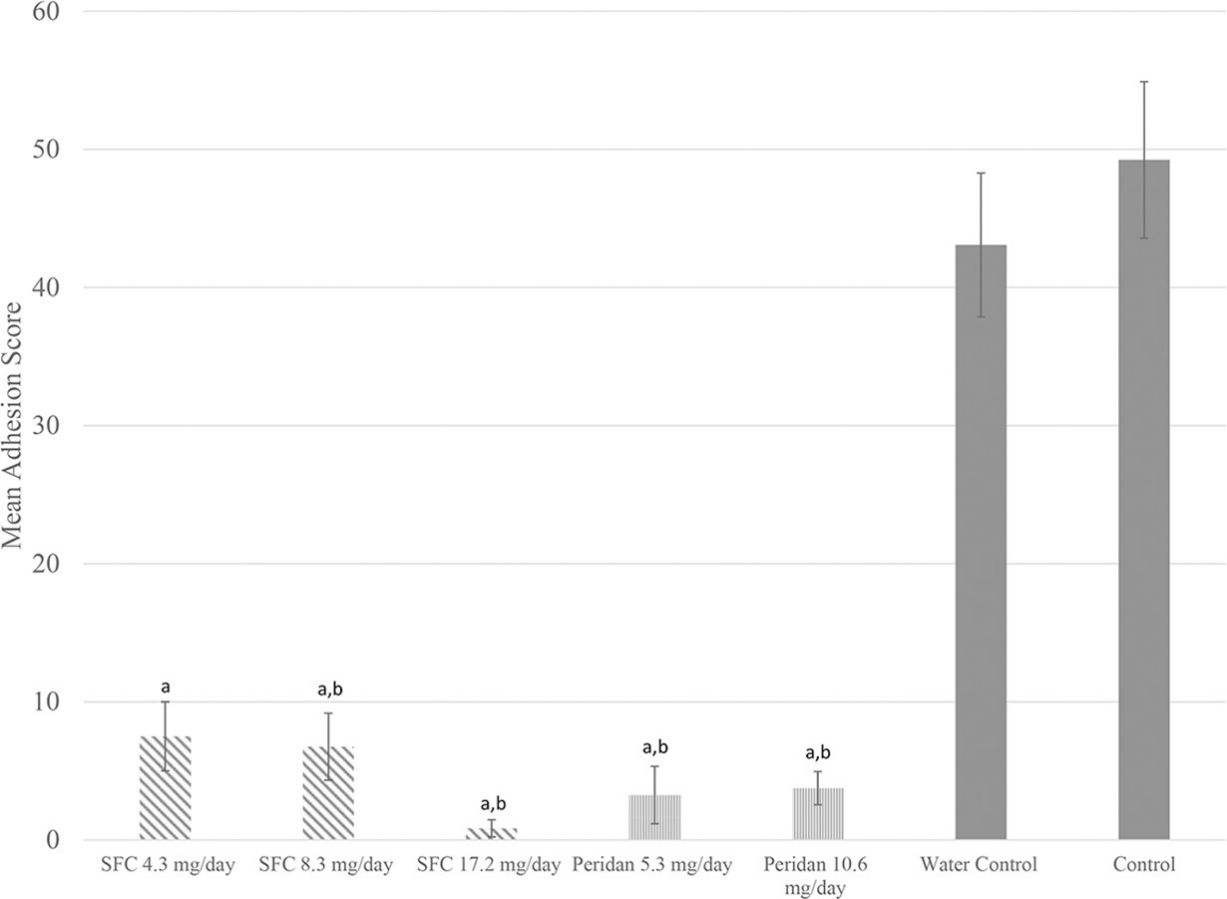
Comparison of mean adhesion score after 7-day continuous IP infusion between experimental groups. Error bars indicate standard error of the mean. a = significantly less than control *p* < 0.05; b = significantly less than water control (*p* < 0.05).

Seven-day continuous infusion of SFC fucoidan at doses of 4.3 mg/day (n = 3), 8.3 mg/day (n = 5), and 17.2 mg/day (n = 6) yielded statistically significant reduction in adhesion scores from water control and no pump controls (Fig 2 and Table 1). Peridan infusion at doses of 5.3 mg/day (n = 5) and 10.6 mg/day (n = 6) also yielded significantly lower adhesion scores as compared to control experiments (Fig 2 and Table 1).

**Table 1:** Effects of varying fucoidan doses on adhesion extent when delivered via subcutaneous pump.

7 Day Continuous Infusion				
Source	Dose (mg/day)	n	Adhesion Score	SEM (+/-)
**SFC**	4.3^a^	3	7.5	2.50
	8.3^a^^,^^b^	5	6.8	2.42
	17.2^a^^,^^b^	6	0.8	0.616
**Peridan**	5.3^a^^,^^b^	5	3.3	2.08
	10.6^a^^,^^b^	6	3.8	1.21
**Control**	Water	13	43.1	5.21
	No Pump	23	49.2	5.67

a = significantly less than control (*p* < 0.05)

b = significantly less than water control (*p* < 0.05)

### Adhesion reduction following bolus injection

**Fig 3:**
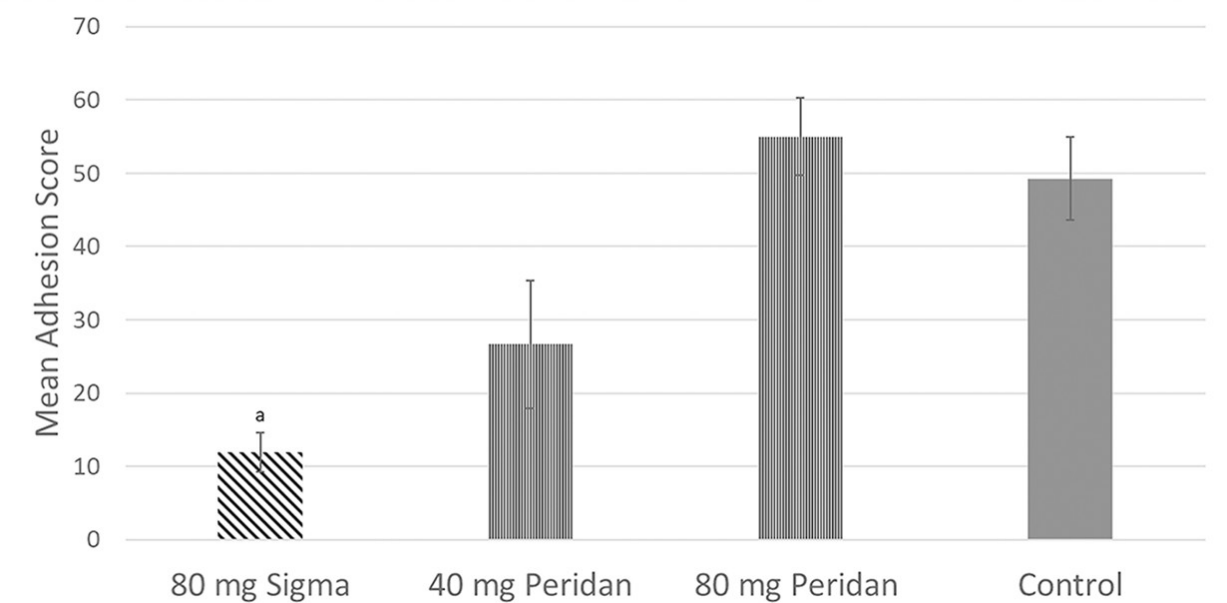
Comparison of mean adhesion score at 7 days post-operation after single intraoperative bolus between experimental groups. Error bars indicate standard error of the mean. a = significantly less than Control group (*p* < 0.05).

Fig 3 shows adhesion scores a week following a single IP dose of 80 mg of either agent (SFC n = 5; Peridan n = 4) and 40 mg of Peridan (n = 3) injected at the time of skin closure. SFC injection led to a substantial reduction in adhesion extent. This effect was not quite as large as that obtained with continuous infusion. In contrast, Peridan given as a single dose had no measurable influence on adhesions in this model.

### Coagulation studies

**Fig 4:**
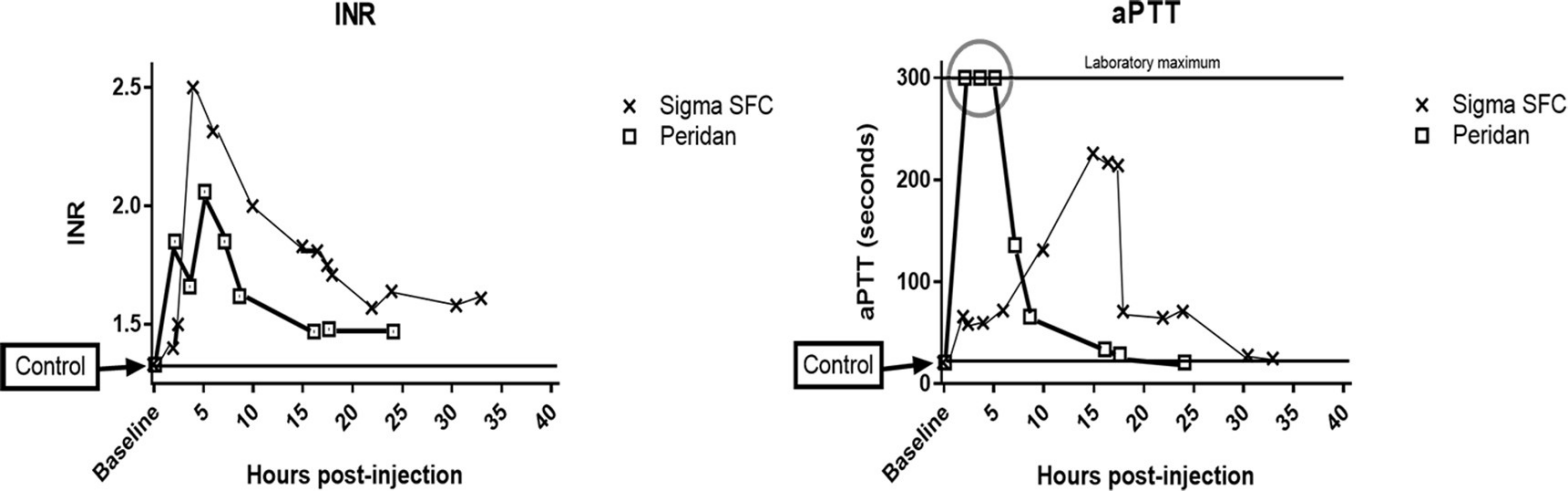
Systemic INR and aPTT at timed sacrifice after IP injection of 50 mg doses of fucoidans. aPTT maximum laboratory reporting value was 300 seconds.

Shortly following intraperitoneal Peridan injection, values of aPTT exceeded the maximum level measured in our laboratory. At 5 hours aPTT was approaching normal and was consistently near control levels after 15 hours. The maximum effects of SFC on aPTT were not reached until 15 hours post injection and fell abruptly thereafter. With either agent, INR values rose only to levels that would be considered therapeutic for human patients being treated with warfarin. These observations are in accord with previous information regarding the anti-coagulant properties of various fucoidan extracts. Activated PTT proved to be the more sensitive measure of systemic coagulation changes (Fig 4).

By contrast systemic INR and aPTT values measured at sacrifice after a week of continuous infusion of similar total doses of the two fucoidans were normal in every instance. Apparently local concentrations at the sites of operative injury are enough to inhibit fibrin clot formation, but the slow constant infusion does not alter systemic coagulation parameters. Regarding the issue of potential bleeding, using fucoidan doses sufficient for anti-adhesive effects, there was no evidence of intraperitoneal bleeding apparent at the time of sacrifice.

## Discussion

Our studies in a rat model have focused on pharmacologic fluid administration to protect the entire abdomen rather than on site specific mechanical barrier agents. Over a number of years, we tested 40 agents administered in fluid form, either as a single bolus dose at the end of the operative preparation, or as a one-week continuous IP infusion from a subcutaneous osmotic pump. Most of these compounds were examined because they had previously been reported to have anti-adhesive capabilities. Very few had been evaluated by means of prolonged continuous infusion. We did indeed observe modest adhesion reductions with a number of these fluid borne agents. In general, the results with one-week IP continuous infusions were more effective than single bolus delivery.

For differing reasons, all except fucoidans fell short as potential candidates for clinical use. Some had no detectable anti-adhesive effects in our model. Others resulted in statistically significant reductions in adhesion formation but of insufficient magnitude to warrant clinical consideration. A few proved to be toxic, or even fatal, when delivered into the peritoneal cavity.

Fucoidans are a class of fucose rich, sulfated, branched polysaccharides derived from various species of brown seaweeds. Among the many fucoidan extracts there are extensive variations with respect to constituents and related biologic activities. Structural, chemical and functional differences between fucoidan extracts depend on the specific seaweed source, extraction methods, and degree of purification. These structural variables are reflected in differing pharmacologic functions [23, 24, 25, 26, 27]. Fucoidans, as a group, have a broad spectrum of biologic activities.

Information regarding parenteral administration of fucoidans in the human is not available. No such product has satisfied the requirements needed to obtain FDA approval for human use. A great deal of effort has been expended to develop clean, consistent extracts suitable for systemic human usage, thus far without success. Fitton [24] has suggested that the production of well-characterized reproducible fucoidan fractions is within reach, and that therapies from fucoidans are a realizable goal. For present considerations, the most pertinent biologic capabilities of fucoidans are anti-inflammatory, anti-coagulant, anti-fibrotic and adhesolytic. Each of these latter processes plays a role in the genesis of abdominal adhesion formation.

To date, information regarding systemic effects of fucoidans has been derived entirely from studies in experimental animals using intravenous or intraperitoneal injections. Our observations of elevated systemic coagulation parameters shortly after IP administration of Peridan or of SFC confirm rapid trans-peritoneal systemic absorption. Intraperitoneal delivery also causes local topical effects. Fucoidan concentrations in or on the peritoneal surface, the locus of adhesion formation, are no doubt far higher than what could be attained with delivery via the systemic circulation.

### Timing

With respect to the time course of post traumatic adhesion formation, we previously reported sequential laparoscopic observations in this rat model [28]. Gross adhesions were seen within 24 hours of operation. Additional adhesions continued to form for up to a week, but no new ones were seen to develop thereafter. Sequential biopsies of the surgically injured surfaces were examined by means of scanning electron microscopy. Plentiful new mesothelial cells were identified on such surfaces by the third day. By seven days, the defect was completely carpeted with mesothelial cells. Thus, surface healing coincided with cessation of new adhesion formation. Subsequent laparoscopic studies in the same animals, weeks and months later, showed adhesion extent and distribution identical to that seen at one week. Our observation that the adhesion process starts immediately after injury and is complete within 7 days is supported by a number of other reports [29, 30].

An important practical principle suggested by these findings is that an antiadhesive agent should be introduced into the abdomen near the end of the operative procedure. A second is that the preventive effects must continue, possibly for a full week.

The progression of adhesion formation is time-dependent. The normal peritoneal fibrinolytic capability is promptly depressed in association with operative trauma. Human and experimental animal studies of peritoneal tissues or of fluid have shown rapid reduction in tissue plasminogen activator (tPA), accompanied by an increase in plasminogen activator inhibitor (PAI-1) associated with operative injury, thereby disrupting the normal ability to lyse newly formed fibrin clots [31, 32, 33]. These circumstances, present at the outset, are optimal for fibrin clot-matrix formation. They revert toward normal in succeeding days.

### Anticoagulants

Historically, many attempts to avert postoperative abdominal adhesions were based on inhibition of clot formation by means of local anticoagulant applications aimed at blocking conversion of soluble fibrinogen to fibrin. This concept continues to find favor today and is likely a central factor for our observed efficacy of fucoidans. Through the years virtually every newly developed anticoagulant agent has been tested with the goal of preventing abdominal adhesions. Much of the early interest in fucoidans was based on the idea that they might provide a substitute for heparin. They have been thoroughly studied and found to be effective anticoagulants [26, 34].

Intraperitoneal use of anticoagulants for adhesion prevention has not proved to be clinically applicable partly because of inconsistent adhesion effects or, in some instances, excessive bleeding [35, 36]. Inescapable facts are that conventional anticoagulants act briefly, but adhesions continue to form for a week after injury. Meaningful adhesion reduction with a single topical application at the outset is not to be expected.

### Thrombosis versus clot

Animal experiments have established important distinctions between hemorrhagic and antithrombotic pharmacological drug effects [37, 38, 39]. Studies in rat and in rabbit models of thrombosis prevention have examined bleeding times and systemic coagulation parameters. Bleeding times were only slightly affected by systemic fucoidan, given in doses which prevented or delayed thrombus formation. On the other hand, doses of heparin sufficient to avert thrombosis lead to a doubling of bleeding time. The investigators estimated that, on a weight basis, fucoidan had 20 to 30 times less anticoagulant effect than did heparin. In terms of anti-thrombosis efficacy versus bleeding risk, fucoidans are measurably superior to conventional anticoagulants, specifically to heparin. In the present studies, we observed that appropriate doses of slowly infused intraperitoneal fucoidans induced major adhesion reductions without causing problem bleeding. But, when the effective total dose was raised by a factor of four or more, we saw major bleeding.

These observations relate to the role of timing for preventing abdominal adhesions following operative trauma. For optimal results, the anti-thrombotic agent is best applied early. This, however, is when the surgically damaged small blood vessels, recently clotted, might resume bleeding when exposed to anticoagulants. In the present studies, such bleeding was not induced by fucoidans given in doses sufficient to inhibit adhesions. This efficacy might be explained by the fortuitous combination of fucoidan biologic capabilities. Anti-inflammation depletes the amount of the fibrin-rich exudate. Prevention of soluble fibrinogen conversion to insoluble fibrin clots, anticoagulation, plays a key role. Antifibrosis delays maturation of fibrin clot to collagen, thereby extending the time available for lysis to take place. In addition to their anticoagulant properties, fucoidans have been shown to have fibrinolytic activity [40].

Adhesion reduction with Peridan in the present studies was accomplished only with prolonged constant delivery. A single bolus of Peridan, given at the end of the operation, had no effect on adhesion formation. By contrast, SFC, given as a single IP bolus, resulted in adhesion reductions of about 75%. Why the difference? That these two fucoidans are derived from different seaweed species and have different molecular constituents no doubt accounts for their differing behaviors, but the molecular specifics are unknown.

### Infection

As noted above, we studied a third, much more highly purified fucoidan, also derived from *Fucus vesiculosis*. This agent is currently marketed as Sigma Fucoidan 95% (Sigma). The same rat model and protocols were employed. Anti-adhesive effects were similar to those of SFC, with mean adhesion scores of 7.1 (2.7 mg/day), 5.1 (5.3 mg/day), and 2.8 (8 mg/day). However, in a significant portion of these rats (30%) we found and cultured unexplained bacterial infections in the peritoneal fluid (S1 File). This complication was seen only twice in the many SFC experiments and not at all with Peridan. The infections were noted over the course of experiments by decreased rat activity and responsiveness, but they were ultimately identified at sacrifice. The infections were not lethal during the seven-day experiment. The presence of peritonitis obviates rational interpretation of adhesion results. These observations will be the subject of a separate communication, focused on the infection issue. As a practical matter, the threat of peritonitis eliminates this fucoidan from clinical consideration and also raises concerns regarding other fucoidans.

The explanation for a particular fucoidan enhancing infection is not clear. A well-established property of fucoidans, in general, is anti-inflammation. A critical aspect of this effect is selectin blocking on leucocytes. The leucocytes are thereby functionally inhibited from passing into tissue spaces. One relevant experimental observation was made in a model of sterile, chemically-induced peritonitis. There was a dearth of leucocytes in the peritoneal fluid of those animals given systemic fucoidan [41]. A plausible hypothesis for our finding of infections is that peritoneal leucopenia prevailed in the rats that received Sigma Fucoidan 95% and allowed infection by otherwise harmless numbers of translocated bacteria. Others who have studied fucoidans for adhesion prevention have not reported any gross complications [19, 20]

Two reports affirm the safety of Peridan solution given into the horse abdomen during operation [42, 43]. While not convincing as to anti-adhesion effects, these observations in a large mammal have special importance with respect to potential human use. The investigators describe no infections or any adverse effects whatsoever. The possibility that the complication of peritoneal infection might be limited to specific fucoidan preparations and doses will require further study. The Peridan horse studies and our rat studies indicate that certain fucoidans in reasonable doses would likely be well tolerated in humans with regards to potential bleeding, infusion reactions, and healing interference. None of these were seen in past animal studies. However, this can only be confirmed after a singular source of well characterized and clean fucoidan becomes available for further study in both animals and humans.

As for human clinical use, the matter of single bolus dose at operation versus several days of continuous infusion is important. Bolus application of an effective anti-adhesion fluid would encourage relatively routine use. On the other hand, if success requires the use of external portable pump drug infusion for some days following the operation, nuisance factors and additional expense might limit its use to high risk situations. Development of a dependable bioresorbable delayed drug delivery vehicle could avoid the need for prolonged IP infusion and encourage common application.

There are limitations to the presently described study that bear consideration. Firstly, the evaluator of adhesion scores was not blinded. Unblinded scoring is a potential source of bias. However, the scorer had scored thousands of similar experiments prior to the work presented in this manuscript, which favors consistency. Secondly, sample sizes were variable and small within experimental groups. While statistical significance was achieved in many cases, larger sample sizes would decrease the variance and paint a clearer picture of the effect fucoidan preparations had on adhesion formation. Finally, there will need to be substantial follow-up studies to this work to characterize additional effects of adhesion administration in both animals and humans. For example, we do not know if fucoidan or fucoidan complexes are subject to molecular mimicry and the development of immune responses that would cause harm after repeat exposure. This is just one of many potential side effects that cannot be commented on with any certainty due to the limited scope of this study.

## Conclusions

In summary, two laboratories, working independently, have tested a total of more than one-hundred candidate fluid agents as to their capacity to inhibit peritoneal adhesions following abdominal operations. Our lab found that certain fucoidan extracts, applied intraperitoneally, yielded anti-adhesive results superior to the many other proposed agents that were similarly evaluated. What is the explanation? Almost all candidate agents studied over the years were aimed at countering a single aspect of the adhesion process, such as inflammation, thrombosis, or fibrosis. Based on the results of coagulation studies done in our lab, we can surmise that thrombosis is attenuated in the presence of fucoidans. Other studies have shown their anti-inflammatory and anti-fibrotic effects. Together, this combination of effects allows doses sufficient to provide major adhesion reduction but small enough to avoid potential complications.

At present the possibility of clinical use is hindered by two barriers. The first is lack of clean and consistent products that could be considered for human use. The second is the potential for engendering infections. Both issues deserve further investigation.

## Supporting information

S1 File Data collected during studies reported in this manuscript. (XLSX)
