# Correction: Fucoidans inhibit the formation of post-operative abdominal adhesions in a rat model

**DOI:** 10.1371/journal.pone.0211371

**Published:** 2019-01-30

**Authors:** Alex J. Charboneau, Greg Beilman, John P. Delaney

The authors are listed out of order. Please view the correct author order, affiliations, and citation here:

Alex J. Charboneau^1^, Greg Beilman^2^, John P. Delaney^2^

1. University of Minnesota Medical School, Minneapolis, Minnesota, United States of America

2. Department of Surgery, University of Minnesota, Minneapolis, Minnesota, United States of America

Charboneau AJ, Beilman G, Delaney JP (2018) Fucoidans inhibit the formation of post-operative abdominal adhesions in a rat model. PLoS ONE 13(11): e0207797. https://doi.org/10.1371/journal.pone.0207797
